# Parents’ and Children’s (6–12 Years Old) Physical Activity Association: A Systematic Review from 2001 to 2020

**DOI:** 10.3390/ijerph182312651

**Published:** 2021-11-30

**Authors:** Rui Matos, Diogo Monteiro, Nuno Amaro, Raul Antunes, Luís Coelho, Diogo Mendes, Víctor Arufe-Giráldez

**Affiliations:** 1 ESECS-Polytechnic of Leiria, 2411-901 Leiria, Portugal; diogo.monteiro@ipleiria.pt (D.M.); nuno.amaro@ipleiria.pt (N.A.); raul.antunes@ipleiria.pt (R.A.); coelho@ipleiria.pt (L.C.); diogo.l.mendes@ipleiria.pt (D.M.); 2 Life Quality Research Centre (CIEQV), 2411-901 Leiria, Portugal; 3 Research Center in Sports Sciences, Health Sciences and Human Development (CIDESD), 5001-801 Vila Real, Portugal; 4 Center for Innovative Care and Health Technology (ciTechCare), 2410-541 Leiria, Portugal; 5 Faculty of Education, Research Unit of School Sports, Physical Education and Psychomotricity, University of A Coruña, 15008 A Coruña, Spain; v.arufe@udc.es

**Keywords:** parental modeling, childhood, MVPA, co-participation

## Abstract

Worldwide, studies reveal that a significant proportion of adults and children do not meet the recommended guidelines for physical activity (PA). Therefore, it is crucial that proxy determinants for child physical activity enhancement could be identified. Parents have been considered to have a key role in child adherence to physical activity. Thus, this systematic review aimed to identify association between parents’ and children’s PA. The search of scientific papers was conducted from 31 October 2020 until 31 January 2021, on the Web of Science, Scopus, Psycinfo, SportDiscus, and Pubmed databases. The PRISMA protocol was used. Findings indicate a consistent association between parents’ and children’s (6–12 years old) PA. Despite the imbalance of the number of assessed fathers and mothers with the latter clearly overrepresented, a trend towards the same gender dyads on PA significant and positive association (father/son, mother/daughter) was evidenced. Results support the relevant importance of parents’ PA as role modeling (either explicitly or implicitly) for children’s PA. Besides, results revealed the importance of promoting PA in family for the enhancement of children’s PA. Moreover, and given the positive impact of exerting PA with the children on children’s PA, parents should spend more time practicing PA with their children, especially on MVPA and meeting the recommended guidelines for PA. Future studies should highlight the role of mediator variables on this interaction process, extending the knowledge on the contribution of other factors to the requested enhancement of children adherence to PA practice.

## 1. Introduction

Regular physical activity (PA) is essential for humans’ health [1]. For children, it is a pillar for a healthy growth and development [2]. Some studies [3,4,5] revealed that many chronical diseases could be prevented if, along with other healthy behaviors, people, as young as children and adolescents, would engage in PA practices, namely of moderate to vigorous intensity (MVPA). Nevertheless, worldwide, studies reveal that a significant part of people do not meet the recommended guidelines for PA [6], children and youth being no exception [7]. In fact, physical inactivity was one of the biggest scourges in the world, constituting an authentic public health problem [8], and is therefore considered by the World Health Organization (WHO) [9] as the fourth highest risk of mortality in the world. Subsequently, some studies [10] not only confirmed WHO’s concerns [9] but also reported that physical inactivity increased in several countries. This led the World Health Assembly (AMS), in 2018, to approve a new Global Action Plan on PA 2018–2030, aimed at reducing overall levels of physical inactivity in adolescents and adults by 15% by 2030 [11]. In this sense, Bull et al. [7] published the new guidelines for the promotion of PA and reduction of sedentary behaviors for children, adults, the elderly, including new specific recommendations for pregnant women and postpartum women as well as for special populations. In a generalist way, Bull et al. [7] recommend that all adults perform between 150 and 300 min of moderate intensity PA or between 75 and 150 min of vigorous intensity, or an equivalent combination of both per week. Among children and adolescents, an average of 60 daily minutes of MVPA intensity aerobic PA should be performed per week. These new guidelines also recommend muscle and bone strengthening, as well as the reduction of sedentary behaviors, for all age groups, although not having defined a limit for sedentary behaviors.

Regrettably, the most recent global estimates showed that one in four adults (approximately 28%) and more than three quarters (more than 80%) of the adolescents do not comply with the recommendations for the practice of aerobic PA [8,12], as stipulated by the WHO [9]. Thus, there is an urgent need to increase priority and investment for services promoting PA both in the context of health and in other crucial contexts [7]. In addition, these data do not demonstrate any general improvement in the PA levels over the last two decades [7,8,12].

Furthermore, despite the lack of control of some variables and the use of different methods when estimating PA, several studies revealed that PA tracks well, especially on boys, from childhood to adolescence and, at least, to young adulthood [13,14,15,16,17]. Therefore, to instill healthy behaviors and habits from early ages seems to be a compensatory strategy favoring the desired outcomes.

To accomplish this goal, it is crucial to understand the PA correlates in children. Amongst other significant ones, parents are considered important mediators of children engagement on PA [18,19,20]. There is a wide variety of ways through which parents can exert a positive influence on their children’s adherence to PA, either in a more indirect (e.g., acting as role models, own beliefs about PA importance) or direct way (e.g., being physically active with their children’s co-participation, encouraging their practice, giving logistic support like driving them to sport facilities [21,22,23,24,25,26].

The social support provided by significant others, including parents, is a concept that has been encompassed in different theoretical frameworks, such as Social Cognitive Theory [27,28]. It is defined as an action or cluster of actions that help a person adopt or maintain a specific practice, which can occur in different directions: (i) instrumental or direct support (e.g., sharing sports equipment, facilitating transport to local practices, and engaging in physical activities together); (ii) psychological/emotional support (e.g., personal incentives, motivation, and encouragement for practices); and (iii) informative support (e.g., acts of orientation, counseling, and talks about the importance and appropriate ways of engaging in PA) [27,28,29]. This mechanism suggests that the role of social support may partly explain the enhancement of self-efficacy, which in turn could influence the commitment to participate and maintain PA [29]. Thus, while children tend to spend a lot of time with their parents, sharing common contextual environments, it is theoretically expected that parents who engage in PA will endorse this behavior. In fact, there seems to be a tendency for children to adopt behaviors like those of their parents as advocated by the social cognitive theory [27,28], meaning that parents who practice PA could provide social support for their children to adopt this behavior in their daily lives.

### Present Study

The present systematic review focuses on papers where children’s and their parents’ PA was quantitatively measured and reported. The reasons for analyzing this period (i.e., 2001–2020) come from the fact that studies [6,7,8,10,12] have revealed a significant lack of PA worldwide and no relevant improvements in the PA levels over these two decades were identified [7,8,12]. In addition, some reviews have looked at the relationship between parents and child physical activity. However, to the best of our knowledge, it seems that none of them have looked strictly at this period or this age group. Indeed, the decision was to restrict analysis on papers that addressed children aged 6–12 years, i.e., after preschool age and before the adolescent years. In fact, preschool children and the adolescents have characteristics (the former a deep dependency, the latter on a way of distancing from the parents’ influence) that might disturb a clean and focused analysis. Indeed, children normally start participating in PA between the ages of 6 and 12, with the strong support of their parents, who offer them various opportunities for fun through sport and other extracurricular activities which promote their development. According to Côté [30], during this stage children’s participation in playful sporting activities takes place naturally, seeking pleasure in their performance. These results were the basis for what would be called deliberate play [30,31] which characterizes the voluntary nature of participation in informal and adapted games with flexible rules, often monitored only by the children themselves, allowing them to play without major requirements, in any space and with varying numbers of players, ages, or sizes [32]. In adolescence and despite the importance of social support provided by parents, several other aspects may assume a greater importance on adolescent’s PA, namely: support from significant others (especially friends and best friends), mother’s educational levels, and family income [33]. Baring in this in mind, this age group was not considered in present study.

Therefore, the aim of present systematic review is to search for association between parents’ and their children’s (6–12 years old) PA from 2001 to 2020.

## 2. Materials and Methods

The present review followed the recommendations by the PRISMA protocol [34]. In addition, the present review does not have a protocol nor registration number.

### 2.1. Research Strategy

A preliminary analysis of several studies related to the main purpose of present study was conducted, to identify the most appropriate databases and keywords prior to conduct the search. Considering the aim of present study and the range of years included in the present review, several databases to cover the maximum number of papers were used. Therefore, a large search of scientific papers was conducted from 31 October 2020 until 31 January 2021, from five different databases commonly used in a wide range of recent literature, e.g., [35,36]: Web of Science, Scopus, Psycinfo, SportDiscus and Pubmed. The type of document was limited to articles. In addition, the bibliography references were also screened to avoid any potential missing articles. The following keywords were used: “parent*,” “parenting modeling,” “parenting practices,” “parent influence,” “parent support,” “parent encouragement,” “parent involvement,” “Children,” “young child,” “child*,” “Physical activity,” “play time,” “outdoor play,” “leisure activity,”. These were used separately or in different combinations, through the inclusion of “AND” or “OR.”

### 2.2. Inclusion/Exclusion Criteria

To be selected for this review the articles needed to meet the following criteria: (1) no restrictions in terms of studies design were considered; (2) published between January 2001 and December 2020; (3) written in English; (4) articles which measure children and parents’ PA levels, regardless of how physical activity was measured (e.g., accelerometers, questionnaire) or its measurement units (e.g., number of weekly trainings, exercise intensity, number of steps); (5) children aged between 6 and 12 years old or a mean age until 12 years old. The exclusion criteria of studies were: (1) systematic reviews; (2) studies published after January 2021; (3) articles which did not specify children or parent PA levels; (4) children mean age below 6 or above 12 years old or mean age above 12 years old. Firstly, studies were screened from titles and abstracts to analyze their eligibility criteria and any full text was not removed at this stage. Secondly, studies were screened in full to determine that all eligibility criteria were met, and no exclusion criteria were applicable.

### 2.3. Data Extraction

Data was extracted by one of the authors using a predefined checklist and was verified and analyzed by two other authors. The following information was extracted: (1) bibliographic information (authors, year of publication); (2) country of the research (3) study design; (4) participants; (5) gender of the participants; (6) age or class of the participants; (7) aim of the study; (8) theoretical background; (9) instruments; (10) variables (PA); (11) main results; (12) methodology quality score.

### 2.4. Quality of Studies

A checklist created by Downs and Black [37] was used to qualitatively evaluate studies’ methodological content. This instrument consists of 27 questions that seek to determine the study’s quality by having in mind several parameters, including study design, adequacy of statistical procedures, description clarity of the main conclusions. The Downs and Black checklist have been used in the sports science domain, e.g., [38,39], and it is appropriate for evaluation of both randomized controlled and non-controlled trials [40]. Two reviewers analyzed the selected studies, and any discrepancies were resolved by an external reviewer. All reviewers were examined and trained prior to the use the Downs and Black [37] checklist. Cohen’s inter-rater agreement presented good agreement (k = 0.834). In the present systematic review, items 13 (“Were the staff, places, and facilities where the patients were treated, representative of the treatment the majority of patients receive?”), 14 (“Was an attempt made to blind study subjects to the intervention they have received?”), 15 (“Was an attempt made to blind those measuring the main outcomes of the intervention?”), and 24 (“Was the randomized intervention assignment concealed from both patients and health care staff until recruitment was complete and irrevocable?”) were not considered, since they were never scored in the papers under analysis. Therefore, the modified scale had a maximum of 23 points from the original one. Finally, no studies were excluded due to low quality assessment score.

## 3. Results

### 3.1. Study Selection

**Figure 1:**
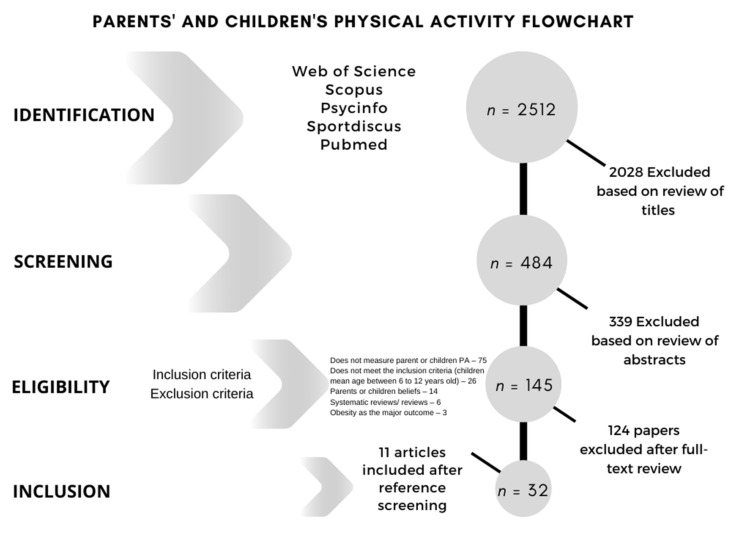
Flow chart of studies.

A total of 2512 titles were identified as potential papers, after checking and removing the duplicate papers from the different databases searched (Figure 1). After a careful read-through of the titles and abstracts of the papers 484 records were screened, with 339 having been excluded for different motives. After the screening phase, the selection was reduced to 145 papers. In total, 124 papers were excluded after the full-text review as they met some of the exclusion criteria. By analyzing bibliographical references, 11 other potentially relevant articles were included, making the total sample of 32 papers that underwent a thorough analysis.

### 3.2. Study Overview Summary

This review includes 32 studies published between January 2001 and December 2020. Table 1 represents a synthesis of the data extracted from the selected studies. The list is organized in alphabetical order by the main author’s name.

### 3.3. Study Characteristics

Table 1 provides a synthesis of the 32 studies included in this review. The majority of analyzed studies were conducted in the USA (*n* = 8) and Canada (*n* = 4). From the 32 studies, 26 were cross-sectional studies, 2 were longitudinal studies, 2 cohort studies, 1 clinical observational study, and 1 quasi-experimental study. In total, 37,960 participants were analyzed (18,693 children and 19,267 parents). Most studies reported children gender (*n* = 28), however, some of the studies did not report parents’ gender (*n* = 18). In those which did report, 51.3% of the children were girls and 68.1% of the parents were mothers. To report PA levels of children, several measuring instruments were used: questionnaires in 17 studies (unique instrument in 15 [25,41,42,43,44,45,46,47,48,49,50,51,52,53,54] and together with a pedometer in two [55,56]), accelerometer in 11 studies [57,58,59,60,61,62,63,64,65,66,67], and pedometer in six studies (unique instrument in four [68,69,70], together with questionnaire in another two [55,56]). To report PA levels of parents, several measuring instruments were also used: questionnaire or survey in 24 studies (unique instrument in 23 [25,41,42,43,44,45,46,47,48,49,50,51,52,53,54,55,57,60,61,62,66,67,71], together with a pedometer in one [56]), accelerometer in five studies [56,57,61,62,63], and pedometer in four (unique instrument in three [68,69,70] and together with questionnaire in one [54]).

Most of the studies’ results (*n* = 25) found a relation between parent’s PA or sport participation and children’s PA level or sport participation [25,41,42,43,44,45,46,47,48,49,50,52,54,56,57,58,59,60,61,62,65,68,69,70,71]. Four studies [51,53,64,67] did not find this relation and the other three [55,63,66] found relations between PA related features (dog walking and PA, parent’s PA and children’s BMI or body fat, and evidence of short co-participation parent/children PA time).

In 20 of the 25 indicated studies, both parents and mothers were found to be present on the PA associations. Of the other five [25,41,42,57,68], only fathers’ PA was found to be associated to their children’s PA in three studies [41,57,68] and only mothers’ PA was found to be associated with their children’s PA in two of them [25,42].

Of the 20 studies where both fathers’ and mothers’ PA was associated to children’s PA, mothers’ PA was more associated than fathers’ in four of them [43,58,69,71]—study [58] found that mothers’ MVPA was found to be related to their sons and daughters’ PA, whilst fathers’ MVPA was only found to be associated to their sons’; study [43] found that the more physically active parents were—especially mothers—the more engaged in organized PA their children were; study [69] found that both fathers and mothers who met the weekend recommendations of 10,000 steps had children more likely to achieve the international weekend recommendations but only mothers meeting weekday recommendations had the same effect on their children; in [70], each 1000 step count increase in mothers’ step count/weekday was associated to higher increases both in sons and daughters compared to fathers’ 1000 step count increase. Moreover, only in mothers was a negative association detected between a 30 min/weekend screen time reduction and extra step count/day on sons and daughters). In one study [59], fathers’ PA was more associated than mothers’ PA to children’s PA, since fathers’ MVPA was associated to sons’ (on weekend and after-school periods) and to daughters’ (on weekdays) MVPA, whilst mothers’ MVPA was “just” associated to daughters’ MVPA (for all time segments), none to sons.

Another addressed issue was the relation of the type of dyads parents/children referring to gender, with the association of parents’ and children’s PA. Eleven studies [25,41,42,44,45,57,58,59,60,61,68] reported an association depending on parents and/or children’s gender. Besides the referred five studies where only mothers’ or only fathers’ PA was related to children’ PA [25,41,42,57,68], 4 of these 11 studies [44,57,58,60] reported associations of fathers’ PA or MVPA with sons’ PA or MVPA but not with daughters. Furthermore, mothers’ PA or MVPA were associated in 4 of these 11 studies [42,44,59,60] to daughters’ PA or MVPA but not to sons’ PA or MVPA. In one of these studies [59], fathers’ MVPA was associated to sons’ MVPA on weekend and after-school periods and to daughters’ MVPA on weekdays, whilst mothers’ MVPA was associated to daughters’ MVPA in all studied periods but never with their sons’ MVPA.

Six studies [43,46,47,59,62,69] reported that having two parents being physically active led to greater PA levels in children than just having one or nonactive parents.

**Table 1:** Synthesis of the selected studies included in the review and their quality.

Author	Design	Country	Participants	Gender	Class/Age	Aim of the Study	Measuring Instruments	Variables (PA)	Results	Studies Quality (Score)
Bois et al. (2005) [25]	Longitudinal study	France	152 intact families (mother, father, and child)	84 girls and 68 boys	From 9 to 11 years of age (M = 9.56, SD = 0.84 years) Their mothers averaged 38.04 years of age (SD = 3.71) and their fathers 39.8 years of age (SD = 4.36)	Mother and father role modelling and parental beliefs in children PA	Perceived Physical Competence Scale for Children Questionnaire on mothers’ perceptions of their child’s physical competence Parents’ PA was measured with a one-week recall format similar to the one parents used to evaluate their child’s physical activity	Child’s physical activity duration Parents’ physical activity	Mothers’, but not fathers’, involvement in PA was related to their child’s involvement in PA	16
Bringolf-isler et al. (2018) [58]	Cross-sectional	Switzerland	889 children and 1059 parents	466 boys and 423 girls 686 mothers and 373 fathers	6–16 years old (10.4 ± 2.6) 227 parents 41 y.old or less; 573 41 to 50 y. old; 76 51 y. old or more	Assess association between children’s and parental PA and whether it varies across different levels of the ecological mode	Actigraph accelerometers models GT1M and GT3X	PA level	Parental (MVPA) was associated with MVPA of their children in general Fathers’ PA was associated with that of their sons, but not with that of their daughters, whereas the association of mothers’ and children’s PA did not depend on the parent–offspring sex-match.	14
Davison et al. (2003) [41]	Cross-sectional	USA	180 children and their parent	Girls	9 year old children Parents were on average in their late 30s or early 40s	Mothers’ and fathers’ parenting strategies and girls’ physical activity; benefit to having two parents who promote and encourage physical activity in comparison with one or no parents	A questionnaire assessing parents’ activity-related parenting practices was developed for the purpose of the study The short version of the Children’s Physical Activity (CPA-short) Progressive Aerobic Cardiovascular Endurance Run	General inclination toward activity, participation in organized sports, and physical fitness Girls’ physical fitness	Mothers provided higher levels of logistic support. Fathers’ behavior encourages activity. both forms of support were associated with higher levels of physical activity among girls	9
Donnelly et al. (2020) [64]	Cross-sectional	Scotland	201 children and their parent		Children (8.41 ± 1.98 years) and one of their parents (38.48 ± 6.91 years)	Explore parent–child associations between these metrics	Anthropometric measures ActiGraph GT3X+ accelerometer	Frequency, intensity	No significant associations	12
Dozier et al. (2020) [48]	Cohort study	USA	132 children and their parent	Child gender Male 65 (49.24%) Female 67 (50.76%) Parents: Male 8 (6.06%) Female 124 (93.94%)	8–12 years old (9.32 ± 0.89 years) Parents 39.11 ± 7.05	Association between parents and children meeting physical activity (PA) guidelines	Parent and child PA data were collected using a survey adapted from the reliable and valid Leisure Time Exercise Questionnaire the child survey included a nine-item scale to assess child perception of parental support	MVPA	B = Boys whose parents met PA guidelines had 3.8 times greater odds of meeting PA guidelines	22
Dunton et al. (2012) [63]	Cross-sectional	USA	291 parent-child pairs	Children were 52.2% female Parents were 87.6% female	Children 8–14 years (11.2 ± 1.5) Mean age of parents was 39.6 years (SD = 6.0 years, range = 26–62 years)	Determine how much time per day that children and their parents spend in physical activity and sedentary behavior together	The Actigraph, Inc. GT2M model activity monitors Portable Global Positioning System (GPS)	(MVPA)	No significant associations	9
Edwardson et al. (2010) [57]	Cross-sectional	UK	117 children	54 boys and 63 girls	Mean age 8.3 ± 0.95	Mothers and fathers’ activity-related support and examine its effect on objectively measured physical activity	A questionnaire adapted from Davison et al. (9) assessing parents’ activity-related parenting practices was completed by both parents Actigraph GT1M accelerometer for 7 days	Moderate physical activity (MPA) and vigorous physical activity (VPA)	Explicit modeling from fathers was found to be associated with boys’ MVPA and VPA	9
Erkelenz et al. (2014) [43]	Cross-sectional	Germany	1615 German children	50.3% male	7.1 ± 0.6 years	Associations between parental PA and children’s BMIPCT, as well as MVPA and participation in organized sports	Children’s MVPA (Opper et al., 2007) parental questionnaire Parents were asked whether they classify themselves as physically active or not (yes or no)	Children’s MVPA Parents PA	Physically active parents had significantly more often children engaging in organized PA than inactive parents	13
Griffith et al. (2007) [55]	Clinical observational study	USA	109 children of 88 parents	55 boys, 54 girls 91.7% were mothers	10–14 years old (mean age 12.0 years)	Determining the extent to which children perceive that their parents influence their day-to-day physical activity	The parent–child dyad completed Baecke physical activity questionnaires Children’s PA—Yamax SW-200 Digi-Walker step counter	PA level	Children who had role models were less likely to be at-risk of overweight. 80% of girls and 67% of boys believed that their mothers would be beneficial in helping them start an exercise program	13
Horodyska et al. (2017) [49]	Longitudinal study	Poland	Children N = 922 Parents N = 922	Children 52.2% girls and 47.8% boys. Parents 16.1% men, 83.9% women	Children were 6–11 years old (M = 8.42, SD = 1.26) Parents were 23–69 years old (M = 35.97, SD = 5.56	Explaining child body fat and PA in a prospective study accounting for data from parent–child dyads	Self-report physical activity questionnaire developed by Godin and Shephard (1985)	Physical activity of parents and children	Higher parental PA (T1) was related to lower body fat in children and PA (T2) on obese children. Parental PA (moment 1) predicted child PA (moment 2)	20
McMinn et al. (2008) [65]	Cross-sectional	Denmark	397 children	Grade 3 = 46.9% were male	Grade 3 (8–10 years) (9.7 ± 0.4)	Associations between sociocultural factors) and objectively measured physical activity in Danish children	Physical activity was measured using a hip-worn MTI accelerometer	PA level	For Grade 3 participants, associations were found between physical activity and parental participation, exposure to sedentary activities, and mothers’ physical activity	15
Mutz and Albrecht (2017) [62]	Cross-sectional		150 pupils and their parents	The sample consists of 80 boys (53%) and 70 girls (47%)	6 and 11 years (M = 8.23, SD = 1.24, Min = 6, Max = 11)	Inner-familial transmission process through which social inequalities translate into differences in MVPA levels in children	Children wore a triaxial accelerometer (ActiGraph GT3X+) Parents sports activities were assessed via questionnaire	PA levels	MVPA in children increases the more sport activities are pursued by the parents. If both parents exercise “more than 4 h per week” the MVPA level of the child is estimated to be 10.9 min/day higher compared to a child whose parents both exercise “less than 30 min per week”	15
Rodrigues et al. (2018) [44]	Cross-sectional	Portugal	834 parents and their children	Girls—424; boys—410	Aged 6–10 years	Associations between children’s participation in extracurricular sport and parental engagement in general or organized PA	Parental questionnaire (To assess parents’ PA and children’s participation in extracurricular sports	Parents’ PA and children’s participation in extracurricular sports	Among girls, the best predictor for participation in more extracurricular sports was having a mother who reported to engage in regular organized PA, while for boys the type of PA practiced by the mother or father was not significantly associated with participation in sports.	13
Saavedra et al. (2014) [68]	Cross-sectional	Spain	1021 subjects (351 children and 670 parents)	Boys—171Girls—180	8.73 ± 0.69 years	Associations between the daily PA of children and their parents	Pedometer Yamax Digiwalker SW200 (for children and parents)	PA levels	The results indicate that the father’s PA is a predictor of the child’s daily PA the role model of a physically active parent, especially the father, positively influences the child’s PA habits	16
Schoeppe et al. (2016) [50]	Cross-sectional	Germany	737 children	51.9% were boys48.1% were girls	10–13 years old (11.0 ± 0.6 years)	Associations between maternal and paternal sport participation, and children’s leisure-time physical activity	Child surveys	Parents Sport Participation Children’s Physical Activity	Higher maternal and paternal sport participation was associated with children spending more minutes per week in leisure-time physical activity	12
Spink et al. (2008) [51]	Cross-sectional	Canada	272 parents and their children	Does not report	5–16 years old 9.25 years (SD = 2.22)	Parents’ level of physical activity would moderate the relationship between parental use of social influence tactics and their child’s physical activity levels	Godin Leisure-Time Exercise Questionnaire	Parent physical activity Parent’s report of child’s physical activity	No significant associations	10
Trudeau et al. (2004) [42]	Cohort study	Canada	166 individuals	87 girls 79 boys	10–12 years old	The potential influence of parental PA on the PA of their offspring when the latter had become adults of similar age	Questionnaire (children and parent)	PA level	Female subjects currently performing PA at least three times per week were more likely to have had active mothers	18
Van Allen et al. (2015) [52]	Quasi-experimental study	USA	Participants (N = 93) included children and their parent	56 girls (58.9%) 87 mothers (93.5%)	Children = 11.57 (2.64) Parent = 39.76 (7.33)	Test associations between parent and child PA	Physical Activity Questionnaire for Older Children [PAQ-C]	PA level	Changes in parent PA may play an important role in long-term PA outcomes regardless of whether youths received PA education as part of the active treatment condition	19
Lijuan et al. (2016) [60]	Cross-sectional	China	323 children	172 boys and 151 girls	7–11 years old (M = 9.83, SD = 0.83)	Association among parental (MVPA) and the MVPA of children by gender	Actigraph GT3X activity monitor-children PA The seven-day MVPA recall developed by Sallis and colleagues (1985) was used to measure parental MVPA	Children MVPA Parental MVPA	the explicit modeling and MVPA of fathers were related to the MVPA duration of boys, whereas the explicit modeling of mothers was significantly associated with the MVPA of girls	14
Lau et al. (2015) [61]	Cross-sectional	USA	671 children and their parents	314 boys and 357 girls	Mean age = 11.49 ± 0.5 years	This study examined associations of various elements of the home environment with after-school physical activity and sedentary time in 671 6th grade children	Children’s PA-ActiGraph GT1M and GT3× accelerometer Parents answered questionnaires PA	PA levels—children Sport participation—parent	Parents’ sports participation was marginally associated with girls’ afterschool total physical activity and sedentary time.	13
Loucaides and Tsangaridou (2017) [71]	Cross-sectional	Cyprus	Children—154; 144 of their parents	61 boys and 93 girls and 144 parents (27 mothers and 117 fathers)	11.7 ± 0.6 (children)	To investigate the effect of attitude toward PA, screen time, parents’ socioeconomic status (SES), and exercise habit on PA and body fatness	Children’s PA-DW-200 YAMAX pedometer Parent PA-questionnaire	PA levels	Parents and friends seem to influence children’s physical activity behavior and time spent outside	13
Salmon et al. (2013) [66]	Cross-sectional	Australia	1220 children and 1220 parents	Children (46% boys); 1001 mothers, 184 fathers	10–12-years old	Associations of dog ownership, dog walking, and physical activity (PA) among children and their parents	Parents MVPA was calculated by summing the duration of moderate-intensity and doubling the duration of vigorous-intensity physical activity Children’s physical activity Actigraph, model AM7164- 2.2C	Parents’ Physical Activity; Children’s Physical Activity	Promoting dog ownership and dog walking among children and as a family are potential strategies for increasing PA participation in some families	15
Vainauskas et al. (2020) [53]	Cross-sectional	Lithuania	486 primary school children	240 boys and 241 girls	7–10 years old	Relationships between physical activity of children and their parents	Godin Leisure-Time Exercise Questionnaire-GLTEQ Parents had to answer a questionnaire	PA level	No significant association	11
Stearns et al. (2016) [56]	Cross-sectional	Canada	612 children and 1 of their parents	289 boys 323 girls	7–8 years old	Examine the relationship between pedometer-measured steps/day of parents’ and their children	Steps Count (SC)-T2 pedometers PA questionnaires	Step Counts	Stronger parent–child PA relationships were observed with pedometers compared to questionnaires, which highlights the importance of using objective measures	12
Bélanger-Gravel et al. (2015) [45]	Cross-sectional	Canada	1000 parent-child dyads	Does not report	11.2 ± 1.3 mean age	Identify individual- and family-level predictors of PA among parent–tween dyads	SHAPE questionnaire—children (IPAQ)—parents	PA level	Significant between-dyad variability in PA was observed among parent–daughter dyads but not on parent–son dyads.	14
Fuemmeler et al. (2011) [59]	Cross-sectional	USA	45 families	23 boys and 23 girls 45 mothers and 45 fathers	Girls 10.6 ± 0.63 Boys 10.6 ± 0.76	To examine parent–child activity correlations	MTI Actigraph accelerometer	PA level	Greater parental MVPA was associated with increased child MVPA. In addition, having two parents with higher levels of MVPA was associated with greater levels of activity in children	14
Welk et al. (2003) [54]	Cross-sectional	USA	994 children and 536 parents	505 boys and 489 girls 82% mothers	9.95 mean age	Explain parental influence on children’s physical activity	Physical Activity Questionnaire for Children (PAQ-C) Parent PA-questionnaire	PA level	Low correlation	11
Singmundová et al. (2014) [69]	Cross-sectional	Czech Republic	485 children and 388 parents	268 girls and 258 boys 252 mothers and 156 fathers	Boys 10.57 ± 1.26 Girls 10.44 ± 1.33	To determine whether physically active parents bring up more physically active children	Pedometer—the Yamax Digiwalker SW-200	Step counts	The children of fathers and mothers who met the weekend recommendation of 10,000 steps were more likely to achieve the international weekend recommendation than the children of less active parents	15
Sigmund et al. (2015) [70]	Cross-sectional	Czech Republic	485 children and 388 parents	268 girls and 258 boys 252 mothers and 156 fathers	Boys 10.57 ± 1.26 Girls 10.44 ± 1.33	Determine the relationship between parents’ behavior (step count (SC) and screen time (ST)) and children’s SC on weekdays and at weekends	Pedometer—the Yamax Digiwalker SW-200	Step counts	1000 SC increase in mothers’ (fathers’) SC/weekend day was associated with an extra 523 (386) SC/day in their daughters and 508 (435) SC/day in their sons. A reduction in mothers’ ST by 30 min per weekend day was associated with an extra 494 SC/day in their daughters and 467 SC/day in their sons.	16
Jago et al. (2010) [67]	Cross-sectional	UK	986 children and 539 parents	493 boys/493 girls 352 mothers/187 fathers	10–11 years old	Examine associations between the physical activity, sedentary and TV viewing patterns of 10–11 year old children and their parents	Actigraph accelerometers	PA level	Higher parental TV viewing was associated with an increased risk of high levels of TV viewing for both boys and girls. There were no associations between the time that parents and children spend engaged in physical activity	18
Wagner et al. (2004) [46]	Cross-sectional	France	3437 children/6393 parents	1713 boys/ 1724 girls 3093 father/3300 mother	12.05 ± 0.56	Determine the association between parental PA and that of their children	Modifiable Activity Questionnaire for Adolescents	PA level	Children were more likely to participate in structured PA outside school when both parents practiced sport as compared to neither parent practicing it A greater percentage of adolescents had a high level of sedentary behavior when both parents versus no parents watched television	13
Eriksson et al. (2008) [47]	Cross-sectional	Sweden	1124 children and their parents	571 girls and 553 boys 1124 mothers and 1124 fathers	12 years old	Investigate parent–child physical activity (PA) associations	Parents PA—Baecke questionnaireChildren PA—question	PA level and sport participation	Parents’ PA was strongly associated with their children’s PA	14

### 3.4. Quality of the Studies

The quality of the studies was assessed according to the previously mentioned 23-point checklist. The study with the highest quality was developed by Dozier et al. [48]. The studies conducted by Davison et al. [41], Dunton et al. [61], and Edwardson et al. [57] presented the lowest quality assessment. The quality of studies is presented in Table 1.

## 4. Discussion

The aim of the present systematic review was to understand the associations between parents’ and their children’s (6–12 years old) PA. Therefore, more than seeking for other possible associations or influences from parents on their children’s PA (e.g., giving logistic, psychological/emotional, or informative support), the focus was on the possible influences exerted by parents through their own PA. Thus, the option was to analyze papers where children’s and their parents’ PA was quantitatively measured and reported.

Although only quantitative measures of any form of PA were considered for inclusion on the review, questionnaires (with most of them presenting concurrent validity to more objective instruments like accelerometers on determining MVPA) were used as the solely instrument in almost 47% (15 out of 32) of the studies. Accelerometers and pedometers were used in, respectively, 11 (~34%) and 6 (~19%) studies. Given the lack of control of some variables and the use of different methods and instruments when estimating PA, results and further analysis should be considered cautiously.

The most significant result was that the majority (*n* = 25, ~78%) of the studies found a relation between parents’ and children’s PA, with only four [51,53,64,67] not finding any kind of significant relation between parents and children’s PA. To the best of our knowledge, there were no other systematic reviews conducted strictly on children aged 6–12 years old. Thus, comparing present findings with previous ones may be a challenging task. Nevertheless, two systematic reviews can be mentioned, despite the inclusion of children from 0 to 18 years old. According to Neshteruk et al. [72] findings on a literature review (2009–2015), although centered just on fathers and in children from 3 to 19 years-old, revealed that 52% of the 27 associations analyzed on the 10 studies that met eligibility were significant, indicating a positive, although modest, relationship between father and children’s PA. Ferreira et al. [33], examining studies from 1980 to 2004, reached somehow mixed conclusions. The authors conducted the review separating children (3–12 years old) from adolescents (13–18 years old). Focusing on the former group that overlaps the most with the present one, when the authors examined the relationship between children’s PA levels and PA levels of their parents (not separating those of the father with those of the mother), they found no relevant associations. Interestingly, studies where father’s and mother’s PA levels were separated from each other revealed that father’s PA levels were positively correlated (in 52% of the cases) to children’s PA (the same percentage of Neshteruk et al. [72] review), whereas mother’s PA levels were mostly unrelated.

This apparent difference between fathers’ and mothers’ PA relation with children’s PA deserves further reflection. As revealed in the present review results section, parents’ PA association with their children’s PA also revealed some differences according to parents’ gender. In 20 of the 25 studies (80%) where that association was detected, fathers’ and mothers’ PA were both influential on (or, at least, related to) children’s PA, which differentiates from the findings of Neshteruk et al. [72], as seen. Even so, despite roughly equivalent fathers’ and mothers’ PA contribution to the association with their children’s PA on 15 of those 20 studies, mothers’ PA was more associated than fathers’ in four of the other five [43,58,69,70]—study [58], since mothers’ MVPA was found to be related to their sons and daughters’ PA, whilst fathers’ MVPA was only found to be associated to their sons’; study [43], because the more physically active parents were—particularly mothers—the more engaged in organized PA their children were; study [69], due to the fact that both fathers and mothers who met the weekend recommendations of 10,000 steps had their children more likely to achieve the international weekend recommendations but only mothers’ meeting weekday recommendations had the same effect on their children; study [70], as each 1000 step count increase in mothers’ step count/weekday was associated to higher increases both in sons and daughters than fathers’ 1000 step count increase. Moreover, only in mothers was a negative association detected between a 30 min/weekend screen time reduction and extra step count/day on sons and daughters. In one study [59], fathers’ PA was more associated than mothers’ PA to children’s PA, since fathers’ MVPA was associated to sons’ (on weekend and after-school periods) and to daughters’ (on weekdays) MVPA, whilst mothers’ MVPA was “just” associated to daughters’ MVPA (for all time segments), none to sons’ MVPA. Additionally, this association was detected only on the dyad father/child on three [41,57,68] of the remaining five studies [25,41,42,57,68], and on the dyad mother/child in the other two [25,42]. These results place fathers’ and mothers’ PA on an equivalent platform of possible influence on their children’s PA, even though a large part of the studies samples was constituted, predominantly, as described, by mothers. In future, to get a better picture of fathers’ and mothers’ PA relation to their children’s PA, studies should clearly differentiate their roles and assume it as an independent or, at least, a moderator variable. Nevertheless, and besides the fact that some of the studies, i.e., [41], suggested (although not being the priority focus of the present review) that mothers might exert more influence on the logistic support while fathers might privilege the role modeling), the relation of the kind of dyads parents/children referring to gender with the association of parents and children PA was also analyzed. As reported, 11 studies [25,41,42,44,45,57,58,59,60,61,68] reported an association depending on parents and/or children’s gender. Besides the five studies where just mothers’ or just fathers’ PA was related to children’s PA [25,41,42,57,68], 4 of these 11 studies [44,57,58,60] reported associations of fathers PA or MVPA with sons’ PA or MVPA but not with daughters. Furthermore, mothers’ PA or MVPA were associated in 4 of these 11 studies [42,44,59,60] to daughters’ PA or MVPA but not to sons’ PA or MVPA. In one of these studies [59], fathers’ MVPA was associated to sons’ MVPA on weekend and after-school periods and to daughters’ MVPA on weekdays, whilst mothers’ MVPA was associated to daughters’ MVPA on all studied periods but never with their sons’ MVPA. Altogether, these results suggest that the link between parents and their children are potentiated whenever dealing with a parent and child dyad of the same gender (father/son, mother/daughter). This may happen because fathers tend to exert a more explicit PA modeling with sons, due to social and cultural factors, while daughters are supposed to follow mothers (and, in general, female) ways of conduct, also in the PA domain. These findings are in accordance with Ferreira et al. [33] review results, which revealed that father role models, compared with mothers, seems to be more relevant to childrens’ PA, particularly in childhood. Bélanger-Gravel et al. [45], noting that the association of mothers’ PA to sons’ PA was absent, whilst present to daughters’ PA, argued that this might happen because mothers and sons might not share the same PA patterns. Regarding role modeling, we think it is very significative in the present review, that 12 of these 25 studies (48%) explicitly use expressions like “explicit modeling”, “role models”, or “parental modeling” to justify the positive associations between parents and children’s PA, in accordance with Bandura [27,28] Social Cognitive Theory. In a conceptual point of view, the Social Cognitive Theory postulates a multifaceted causal structure in which self-efficacy beliefs operate together with goals, outcome expectations, and perceived environmental barriers and facilitators in the regulation of human motivation, behavior, and well-being [27,28]. Thus, it is reasonable to assume that social support (logistic or modeling) provided by parents is a crucial determinant to action for children’s PA, since PA has been shown to be modeled by significant others, and these significant others may also provide approval and opportunities for PA. Although parents’ PA is not the only factor explaining children’s PA (e.g., [41] highlighted the importance of the logistic support—of mothers), results show that, at least, parental PA has the potential to act as a proxy for parental PA modeling. Co-participation, i.e., the simultaneous and interactive PA participation of parents and their children, is a particular PA feature that can potentiate children’s PA. This happens because it assures that, during that time, children are enrolled in PA as, also, because it may serve as a more direct feature of parental PA modeling (when not co-participating, children may not be aware of parents’ own PA enrollment). In fact, the expression “explicit modeling” is often used with this co-participation intention [41,45,46,57,60,65,69]. All in all, it seems that parents tend to model the children’s behavior as consequently adopting it as a part of their repertoire. In addition, parents may also become strong supporters for active lifestyles that children could develop across lifespans.

Six studies [43,46,47,59,62,69] reported that having two physically active parents led to greater PA levels in children than just having one or nonactive parents. This may reinforce the importance of parental PA modeling on PA of their children and justify that PA, whenever possible, may be taken under a family context to enhance the positive benefits of explicit parental modeling. This was proposed by Salmon et al. [66], about regular dog walking and its benefits for PA among some families, Sigmundová et al. [69] and Sigmund et al. [70], suggest the weekend as an adequate moment for children PA promotion in families and Eriksson et al. [47], advocating the family as an important target for interventions to increase PA in children.

Finally, and given the fact that studies have shown a substantial part of subjects (children included) all around the world that do not meet the recommended guidelines for PA [6,7], it is worth to point out Dozier et al. [48] results revealing that boys whose parents met PA guidelines had 3.8 times greater odds of meeting PA guidelines. Still about recommended guidelines, Singmundová et al. [69] showed that the children of parents who met the weekend recommendation of 10,000 steps were 5.48 and 3.60 (fathers and mothers, respectively) more likely to achieve the international weekend recommendations than the children of less active parents. Following a similar path, Mutz and Albrecht [62] concluded that if (both) parents exercise more that 4 h/week, MVPA level of the child was estimated to be 10.9 min/day higher, compared to a child whose parents both exercise less than 30 min/week. Furthermore, and since the referred guidelines invoke, for children and adolescents, an average of 60 daily minutes of MVPA intensity aerobic PA per week [7], the results of Saavedra et al. [68] explicitly indicate that father’s PA is a predictor of child’s daily PA. Finally, Sigmund et al. [70] revealed that 1,000 step increments in mothers’ step count/weekday was associated to an extra 523 step count/day in their daughters and 508 step counts in their sons (386 and 435, respectively, with fathers). These are very promising results that reinforce the association between parents’ and children’s PA. Finally, we highlight the conclusions of another systematic review study [73] which confirms that most studies observed a weak positive relationship between the PA of parents and children, regardless the age of children, the gender of the parent–child dyad, and the type by PA.

Future research should clearly address the differentiated role models and relation of mothers’ and fathers’ PA to children’s PA, as well as extend the present analysis to other child ages, namely preschoolers and adolescents. It is also recommended that, in future, more longitudinal studies should be undertaken so that parents’ PA eventual influence on children’s PA can become clearer. It is also recommended that, despite the high correlation and concurrent validity of diverse used instruments like questionnaires, research adopts a more objective quantitative measure of PA and that MVPA and the recommended guidelines for PA may serve as the main PA referential, making it easier to compare of the results of the various performed studies.

### 4.1. Practical Recommendations

Synthesizing previous appreciations and considerations on present results, several practical recommendations deserve consideration. Firstly, fathers’ and mothers’ PA seem to be positively related to child PA. Given the lack of a significant number of longitudinal studies, it is not possible to affirm that parents’ PA influences children’s PA, since the identified relation can derive from an inverse or bilateral reciprocal influence. Therefore, parents should adopt an active lifestyle so that their children adhere the most to PA. Secondly, given that fathers’ PA seems to be more “influential” on boys and mothers’ PA on girls, this should be something that parents should be alerted to. Furthermore, the combined effect of fathers’ and mothers’ PA seem to potentiate children’s PA. Thus, and according to the studies that also revealed the importance of other family members (e.g., siblings and friends) on children’s PA, strategies that promote PA in family should be adopted. Besides this “in family” PA strategy for the promotion of children’s PA, the referred co-participation seems to deserve special attention. Given the revealed importance of parents exerting PA with the children on children’s PA levels [41,45,57,60,65] and the effective (very diminutive) time spent by parents and their children together practicing PA [63], this is a practical recommendation that can be extracted from the present review. Parents should find a way and be supported (e.g., more friendly work timetables, more active utilization of weekend period in family) to spend more time practicing PA with their children, especially on MVPA and meeting the recommended guidelines for PA [6,7].

### 4.2. Limitations

Although the present study contributes to the identification of links between parents’ and their children’s PA, it has some limitations. The present review could not determine (nor was its intention) the reasons that justify the eventually detected associations between fathers’ and mothers’ PA and children’s PA. They may be more indirect (implicit and explicit modeling) or direct (co-practice). As previously mentioned, future studies could try to clearly address the differentiated role models and relation of mothers’ and fathers’ PA to children’s PA.

The present review was focused on children aged 6–12 years old. Nevertheless, by including studies whose subjects mean age ranged between 6 and 12 years old, we did not prevent the possibility of some results’ contamination by subjects that were not the review focus (6–12 years old). Yet, this was a risk conscientiously assumed. Not acting this way might exclude analysis studies that were, in fact, mainly centered on our target ages, even though containing a few subjects of other age bands. Using the mean age as reference was the way we thought there still subsisted a significant portion of subjects within the targeted age range. Moreover, that is something that emerges on “just” 5 out of the 32 studies and, even there, standard deviation is small, which places the majority of subjects on the focused range. Finally, 14 of the 32 studies do not mention the age range but only mean ages. Nevertheless, that is something we assume and, therefore, we are putting it as a limitation of the study, since we cannot be completely sure that some preschoolers or adolescents did not affect, even slightly, some results. Thus, future reviews might address other child ages, namely preschoolers and adolescents. It is also recommended that, in future, more longitudinal studies are undertaken so that parents’ PA eventual influence on children’s PA can become clearer. Despite the high correlation and concurrent validity of diversely used instruments like questionnaires, research adopts a more objective quantitative measure of PA (essentially accelerometers) and that MVPA and the recommended guidelines for PA may serve as the main PA referential, making it easier to compare of the results of the various performed studies.

## 5. Conclusions

Findings from our review indicated a consistent association between parents’ and children’s (6–12 years old) PA. Despite the imbalance on the number of assessed fathers and mothers through the elected studies, with the latter clearly overrepresented, a trend towards the same gender dyads on PA significant and positive association (father/son, mother/daughter) was evidenced. Results support the relevant importance of parents’ PA as role modeling (either explicitly or implicitly) for children’s PA. The importance of promoting PA in a family context for the enhancement of children’s PA, as well as parents spending more time in PA co-practicing with their children, especially on MVPA and meeting the recommended guidelines for PA, also emerged from the studies analyzed.

Future studies should highlight the role of mediator variables (e.g., type of parent’s job, parent’s sport or PA experience, neighborhood environment, transportation to PA place, among others) on this process, extending the knowledge of the contribution of other factors to the requested enhancement of children’s adherence to PA practice.

## Data Availability

Data is contained within the article. For detailed information of each part please contact the corresponding author.
